# Supplementary material for: Video Recording Can Conveniently Assay Mosquito Locomotor Activity
Source: Sci Rep. 2020 Mar 19;10:4994. doi: 10.1038/s41598-020-61733-5 (PMC7081347; doi:10.1038/s41598-020-61733-5)
Supplement: Supplementary file 1 — Supplementary Information. [file 41598_2020_61733_MOESM1_ESM.pdf]

Video Recording Can Conveniently Assay Mosquito Locomotor Activity

Maisa da Silva Araujo[1], Fang Guo[2] and Michael Rosbash*

*Howard Hughes Medical Institute and Department of Biology, Brandeis University Waltham, MA 02454

United States of America

e-mail: rosbash@brandeis.edu

[1] Current address: Laboratory of Entomology, Fiocruz Rondônia, Brazil and PGBIOEXP/PNPD, Federal University Foundation of Rondônia, Brazil

[2] Current address: Department of Neurobiology, Key Laboratory of Medical Neurobiology of the Ministry of Health of China, Key Laboratory of Neurobiology, Zhejiang University School of Medicine, Hangzhou, Zhejiang 310058, China

Supplementary Video. Representative video of mosquito activity monitoring in the 24-well Flybox.
